# A tiled multi-city urban objects dataset for city-scale building energy simulation

**DOI:** 10.1038/s41597-023-02261-5

**Published:** 2023-06-02

**Authors:** Rui Ma, Dongping Fang, Jiayu Chen, Xin Li

**Affiliations:** 1 Department of Architecture and Civil Engineering, City University of Hong Kong, Hong Kong SAR, China; 2 School of Civil Engineering, Tsinghua University, Beijing, China

**Keywords:** Energy management, Energy efficiency

## Abstract

City-scale building energy simulation provides a significant reference for planning and urban management. However, large-scale building energy simulation is often unfeasible due to the huge amount of computational resources required and the lack of high-precision building models. For such reasons, this study developed a tiled multi-city urban objects dataset and a distributed data ontology. Such a data metric not only transforms the conventional whole-city simulation model into patch-based distributed simulations but also incorporates interactive relationships among objects in cities. The dataset stores urban objects (8,196,003 buildings; 238,736 vegetations; 2,381,6698 streets; 430,364 UrbanTiles; 430,464 UrbanPatches) from thirty major cities in the United States. It also aggregated morphological features for each UrbanTile. To validate the performance of the developed dataset, a sample test was conducted in one city subset (Portland). The results conclude that the linear increase of time usage of modeling and simulation with the increase of building numbers. With the tiled data structure, the proposed dataset is also efficient for the building microclimate estimation.

## Background & Summary

In 2015, all United Nations Member States agreed and adopted the goal of “Sustainable cities and communities” as part of the 2030 Agenda for Sustainable Development (https://www.un.org/). This goal emphasizes decreasing energy consumption and carbon emissions of cities and settlements, then finally achieving the goal of carbon neutrality. Buildings, as the major energy consumer in urban areas^1^, have the highest energy-saving potential. To promote building energy efficiency, building energy simulation is a powerful tool to identify proper energy-saving solutions, such as using alternative construction materials^2^ and improving energy management systems^3^. In the context of the city environment, the simulation approach is a significant decision-making reference for local governors and city managers. It can be used for benchmarking energy efficiency, evaluating scenarios, and analyzing peak energy loads and usage patterns^4^. However, complex urban systems pose two major challenges for city-scale building energy simulation. (1) The huge number of buildings in a city requires unaffordable computational resources. The building energy simulation relies on thermal-physical theories and requires a comprehensive computation process for a whole year. Therefore, modern simulation models, such as urban modeling interface (UMI)^5^, City Building Energy Saver (CityBES)^6^, and City Energy Analyst (CEA)^7^, have to scarify flexibility and reliability by simplifying calculation with statistical analysis or reducing the simulation scale to neighborhood or community levels. (2) Existing city digital data formats, such as GeoJSON, Shapefile, and CityGML, do not provide sufficient information to infer spatial connections and inter-building effects. While some administrative entities, such as the governments of New York City (https://data.ny.gov/) and Portland (https://www.portland.gov/omf/bts/cgis), are making simple building information available online, these datasets only include basic geometric information and lack a hierarchical structure suitable for city-scale simulations. Some researchers, like Chen and his colleagues, have attempted to refine urban building information by adding more detailed energy consumption data^8^. However, even with these refinements, the information provided only covers geometry and building attributes, and does not include descriptions of the surrounding environment. This missing piece is crucial for assessing building energy consumption, as it directly affects heat transfer and radiation reception^9^.

To assess the interactions (such as heat exchanges and radiation absorption) among urban geographic objects (such as buildings, trees, and water bodies), the city-scale simulation requires importing all digitized objects together into the simulation engine. However, as whole city simulation requires formidable computing resources, it is impractical in a real urban scene. Researchers proposed an alternative solution by simulating individual buildings’ energy performance with surrounding environments’ morphological features one by one and then aggregating the equivalent results into a whole city^10^. The surrounding environment is a compulsory input for various simulation functions, such as the evaluation of inter-building effects, microclimate estimation, etc. However, this method requires specifically designed datasets for ease of implementation and calculation. In addition, such a dataset should incorporate both each building’s digital model and its associated surrounding environment. To fill this research gap, this study developed a tiled multi-city urban object dataset for 30 cities. Also, this study proposed a novel tiled data structure based on ontology theories. The designed ontology format allows extracting the tiled data from geographic information systems and existing digital building models and leverages the simulation efficiency with parallel and distributed computation mechanisms.

## Methods

### Fundamental architecture of the dataset

The developed dataset includes two sets of data, including the semantic building information and the surrounding physical object information. Such a setting intends to accommodate to model input structure of thermal-physical-based building energy simulations. The semantic information is used to construct buildings’ physical and geometrical models, and surrounding objects are used for thermal dynamic environment assessment. To properly design the architecture of the dataset, this work utilized the Resource Description Framework (RDF) graphs to represent the qualitative relationships among objects. To construct a suitable RDF graph, the data ontology should be properly defined. An ontology is a formal, explicit specification of a shared conceptualization^11^. It can be used to encode knowledge for sharing, integrating, and linking data from different domains. In general, an ontology consists of classes, individuals, and properties. Classes can be interpreted as sets that contain individuals; individuals are the “instances of classes” and encode fundamental information; properties are binary relations of individuals. This study used Web Ontology Language (OWL) as a vocabulary extension of RDF for ontology development.

This study chose to use RDF, OWL, and other semantic web technologies to build an ontology-based dataset instead of a traditional relational database because ontologies adopt Open World Assumption (OWA), while relational databases are based on Closed World Assumption (CWA)^12^. The most significant difference between the two is their understanding of things that are not explicitly declared. In a relational database, if an entity does not have any relationship declaration, the search result will be an empty set. However, an ontology can infer hidden information based on other known declarations, making it more suitable for extracting surrounding environment information of the target UrbanTile/building during the distributed simulation period. Furthermore, external RDF data can be linked through predefined relations^13^, enabling the integration of data from different fields, making the dataset usable for various applications such as district energy management^14^, building life-cycle decision-making^15^, IoT- and cloud-enabled smart communities or cities^16^. However, creating vocabularies and rules for ontology definition can be a disadvantage of using an ontology. Additionally, the performance of the ontology is affected by the scale and quality of vocabulary, and users need to have a well understanding of the ontology structure and query logics. Despite these drawbacks, the ontology-based dataset offers significant benefits in terms of communication, interoperability, and information inference.

The tiled data structure proposed by this study is called the UrbanPatch Topology Ontology (UPTO). The UPTO encodes building geometric semantics and spatial features into three levels.

- Level 1 - Objects. Objects are initial and original classes, including the semantics of Building, Vegetation, and Street. These objects can be accessed from various public sources, such as OpenStreetMap. Objects are typical individual classes that have no spatial semantics between each other and are spatially discrete.
- Level 2 - UrbanTile. UrbanTiles encapsulate all objects that are separated by natural or artificial boundaries. For example, a small community (including all buildings, vegetation, and waterbodies within it) that is separated from the local urban region by roads is a typical UrbanTile.
- Level 3 - UrbanPatch. UrbanPatch is the geographical boundaries of local microclimates for a target building or a UrbanTile. In general, an UrbanPatch contains the spatial semantics of the target building/UrbanTile and its surrounding classes. These classes can be building, vegetation, street objects, or other UrbanTiles.

**Fig. 1:**
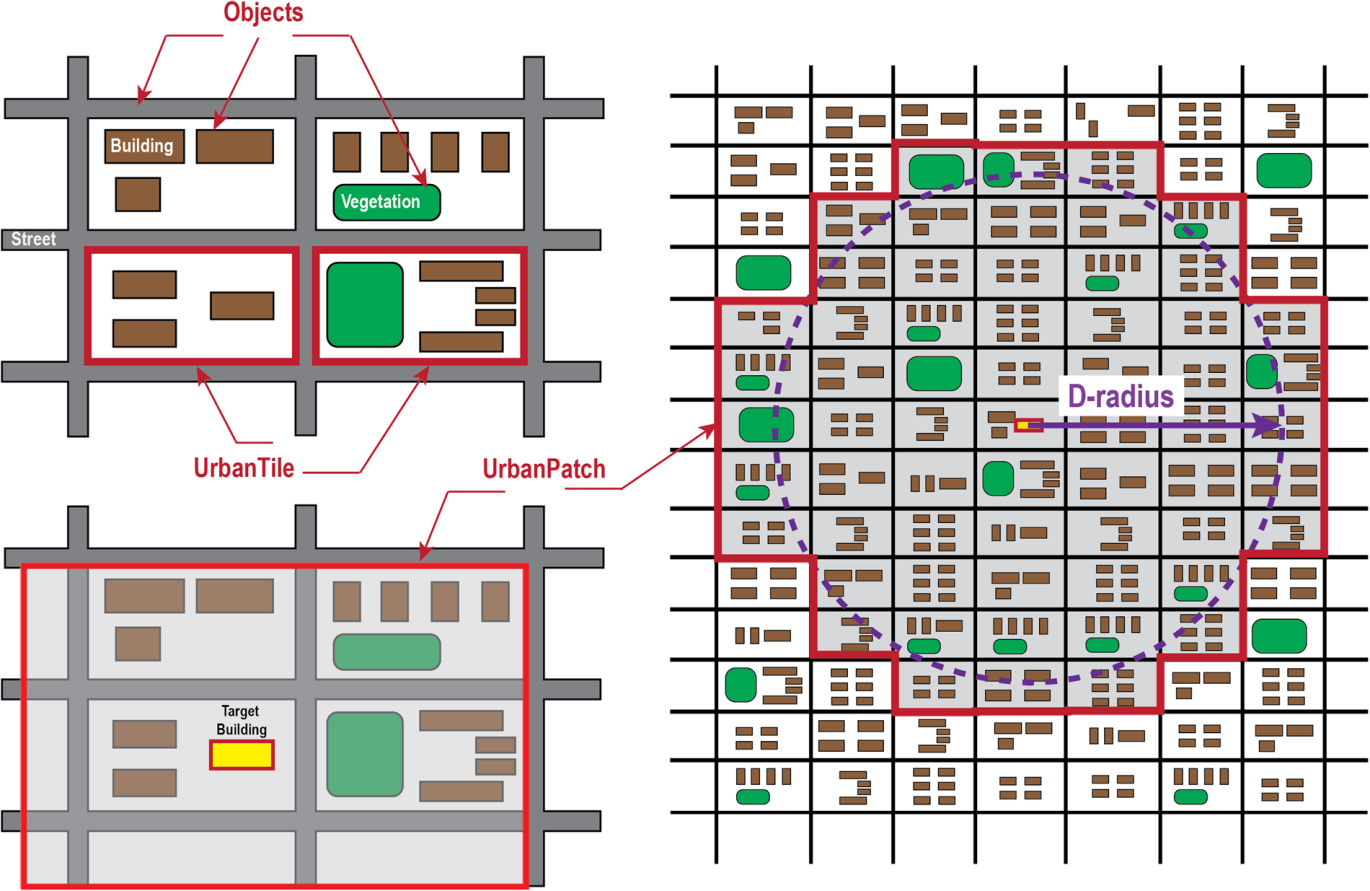
Three class levels of UPTO.

Figure 1 shows the three class levels of UPTO. It is worth mentioning that the UrbanPatch is an object-dependent class, and it can specify the surrounding environment of a building or a UrbanTile. The major difference is if the UrbanTile that a building belongs to will be regarded as part of UranPatch. When using UrbanTile as the UrbanPatch target, the dataset size can be greatly reduced, but all objects in the same UrbanTile will share the same UrbanPatch as its surrounding environment. Smaller file/data sizes can improve the efficiency of the simulation but losses precision.

### UrbanPatch topology ontology

**Fig. 2:**
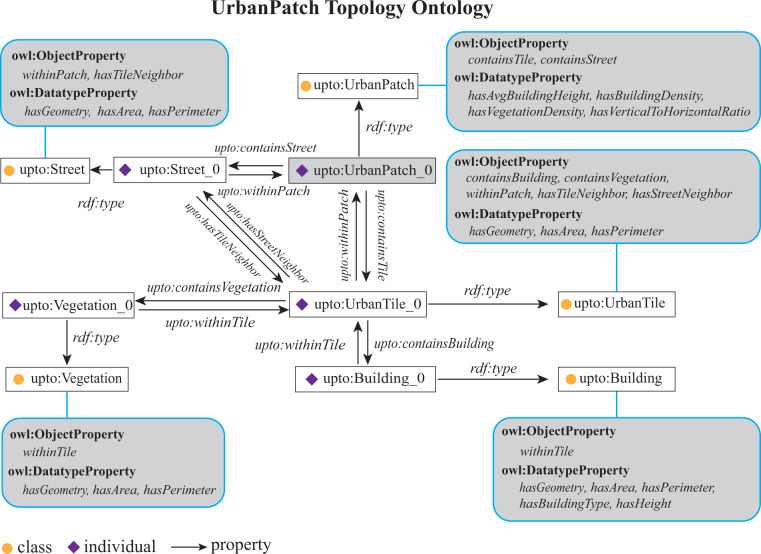
The Semantic structure of the UrbanPatch Topology Ontology.

Figure 2 shows the proposed semantic structure of UPTO, where each UPTO class has predefined Object and Datatype properties. An object property is defined as an instance of the built-in OWL class “owl:ObjectProperty”, representing the spatial relationship between individuals. A datatype property is defined as an instance of the built-in OWL class “owl:DatatypeProperty”, referring to the corresponding morphological features. As shown in the figure, “rdf:type” refers to a resource as an instance of a class. Using the UPTO to define building semantics has three advantages. First, an ontology can explicitly define internal classes and their relationships. It can remove the definitional ambiguity of internal items. Second, the ontology provides a spatial semantic context for each individual building, which helps to form a linked semantic graph. Spatial connections or features can be calculated with rule-based or other computational reasoning. For example, when the “containsTile” property of “UrbanPatch_0” points to “UrbanTile_0” and “containsBuilding” property of “UrbanTile_0” points to “Building_0”, it can be inferred that “UrbanPatch_0” contains “Building_0” in the physical world. Third, with a well-structured semantic structure, the ontology can be easily extended with other external ontologies. This advantage is especially useful when data requires to have high interoperability.

#### Level 1 – Object

A building, vegetation, or street can be defined as an object. Object class has the data property of “hasGeometry”, “hasArea”, “hasPerimeter”. Based on such a predefined structure, each object in a city can generate an instance by filling the raw geometry. These raw geometries can be accessed from different sources, for example, they can be downloaded from the OpenStreetMap with the Overpass API (https://wiki.openstreetmap.org/wiki/Overpass_API). The key of raw geometries should be properly mapped to UPTO properties. For example, this study utilized OpenStreetMap’s closed polygons with the key of “building” as the raw geometry inputs. Also, for different data sources, the same object may be tagged with different key values. Also, take the OpenStreetMap dataset as an example, all relevant keys for vegetations include “natural” = “wood”, “natural” = “scrub”, “natural” = “wetland”, “leisure” = “park”, “leisure” = “garden”, “leisure” = “pitch”, “leisure” = “playground”, “landuse” = “grass”, “landuse” = “farmyard”, and “landuse” = “meadow”. The building object has a significant property name of “hasBuildingType” (with the value of “Office”, “School”, etc.). This property is crucial to infer a building’s semantic data and more comprehensive features, such as thermal zoning, construction, material, etc. Due to the lack of full building information models, the missing information of a building can be inferred with predefined generic models (https://www.energycodes.gov/prototype-building-models).

**Fig. 3:**
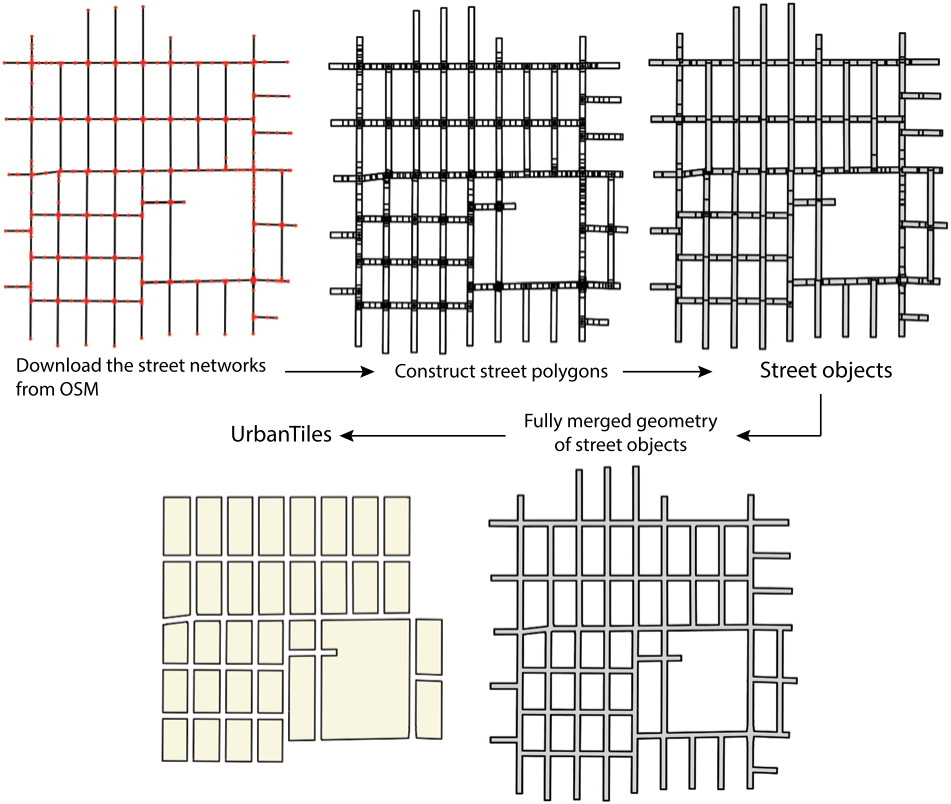
Generation process of street objects and UrbanTiles.

To compensate for the lack of sufficient street width in the OSM database, it is necessary to generate street objects using the steps outlined in Fig. 3. First, the street networks should be downloaded by querying the “highway” key with a “value” that corresponds to the size of the patch enclosed by the street. Previous research by Huo *et al*. has shown that patch sizes ranging from 200 m to 2 km achieve valuable results for modeling urban thermal environments^17^. In this study, the “value” was chosen from a selection of options including ‘primary’, ‘secondary’, ‘tertiary’, and ‘residential’, and the patch size was restricted to a horizontal distance ranging from 200 m to 500 m. The street networks are composed of line segments and are formatted as Shapely.Geometry.LineString objects (https://shapely.readthedocs.io/). The next step involves constructing street polygons with a default width of 3 m for a single lane. Street polygon outlines can be generated using the Shapely.Geometry.buffer function, and street objects can be created by inputting these outlines into the Shapely.ops.polygonize function. This approach enables the generation of street objects with sufficient width to model the urban thermal environment accurately.

#### Level 2 – UrbanTile

A UrbanTile is a class to represent a collection of multiple objects enclosed by its spatial boundaries. The UrbanTile is designed to link the discrete city objects (streets, buildings, and vegetation) based on their geographical locations. Each object associated with a UrbanTile will have its property “withinTile” updated, whereas the UrbanTile will also update the property “containsBuilding”. Also, “hasStreetNeighbor” and “hasTileNeighbor” are such interrelated properties that should be updated for both the objects and UrbanTiles. Figure 3 illustrates the process involved in generating UrbanTiles. The first step is to merge all the previously generated street objects into a single layer. In this study, street objects and UrbanTiles are considered complementary in the 2D urban plane. The areas enclosed by the merged streets are therefore regarded as UrbanTiles. The merged streets can be visualized as a network of lines defining the edges of the UrbanTiles. This process ensures that UrbanTiles are accurately delineated and closely aligned with the underlying street network. By providing a consistent and standardized representation of urban areas, the UPDS dataset can facilitate more effective energy management strategies and support the development of sustainable urban environments.

#### Level 3 –UrbanPatch

The morphological conditions that surround a building not only determine its local microclimate conditions but also have comprehensive thermal exchange properties. The UrbanPatch class is designed as the perception domain of microclimates for a class instance. The class instance can be an object or a UrbanTile, and the selection of an object or UrbanTile determines the precision of the assessment. In addition, the size of UrbanPatch can be adjusted by a parameter D-radius. According to Oke’s research^18^, a rule of thumb of a 500 m radius is sufficient to estimate the local thermal exchange. As shown in Fig. 1, the D-radius determines how many adjacent UrbanTiles will be included in a UrbanPatch. These adjacent UrbanTiles are stored in the properties of “containsTile” and “containsStreet”.

**Fig. 4:**
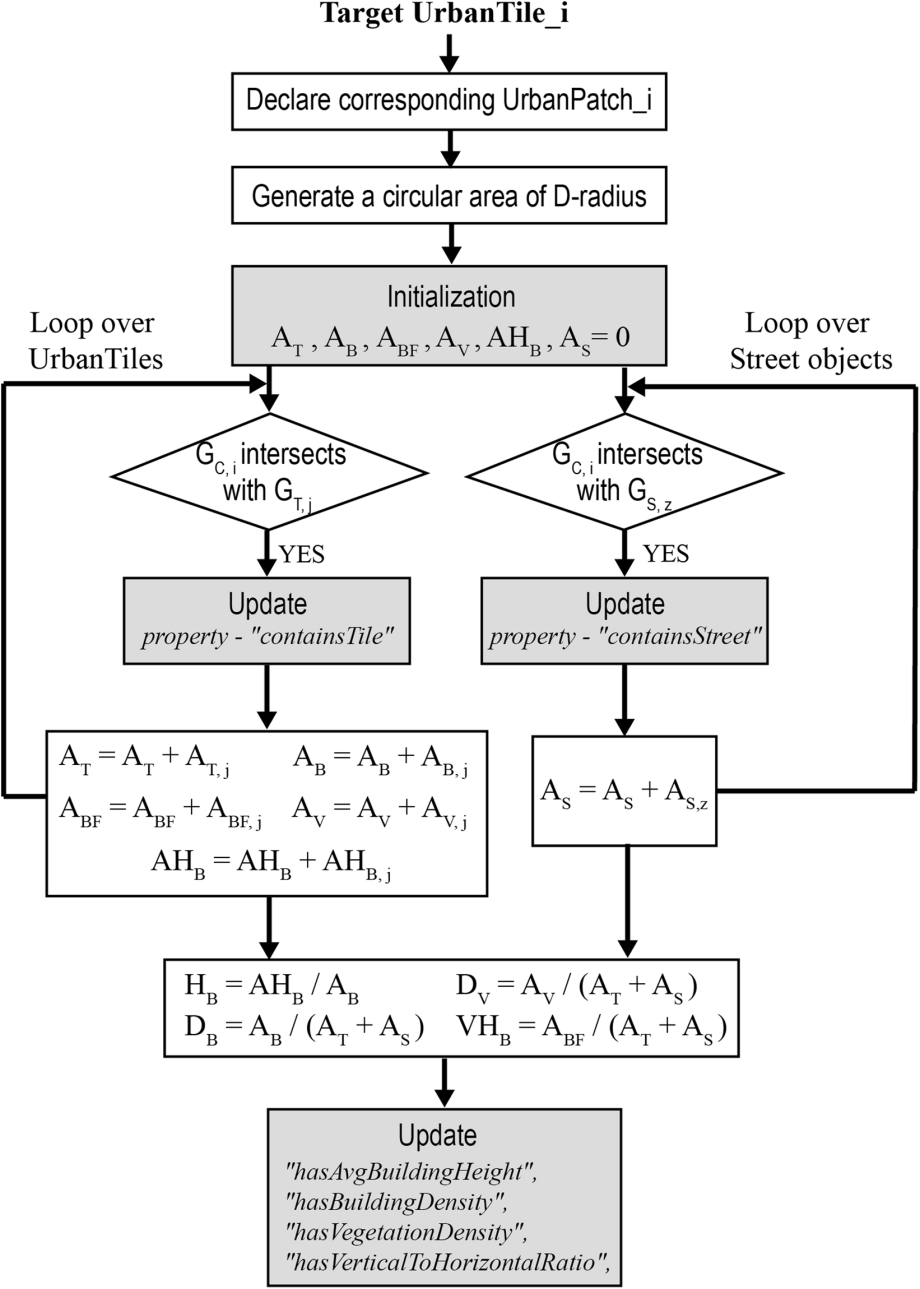
Computation flow of the UrbanPatch instance.

Object classes encompass raw building geometry information, whereas UrbanTile and UrbanPatch contain derived properties of building geometries and morphological features. Assuming there is a UrbanTile_i, Fig. 4 shows the workflow of filling the information of UrbanPatch_i based on UPTO. Table 1 lists the variables and their description of constructing a UrbanTile instance. An UrbanPatch can be considered as the combination of the internal UrbanTiles and boundary street objects. The stored information is not the original UrbanTiles and objects’ content, but the reference ID of instances. The datatype properties also stored the aggregated morphological features, such as *H*_*B*_*, D*_*B*_*, D*_*V*_*, VH*_*B*_, etc. These morphological features are crucial in tuning the typical meteorological year (TMY) weather data into local microclimate conditions with Urban Weather Generator (UWG)^19^.

**Table 1:** Variables used in UrbanPatch Construction.

Variable	Description	Variable	Description
*A*_*T, j*_	Footprint area of UrbanTile_ j	*A*_*T*_	Sum of *A*_*T, j*_ at the patch scale
*A*_*B, j*_	Footprint area of the internal buildings of UrbanTile_ j	*A*_*B*_	Sum of *A*_*B, j*_ at the patch scale
*A*_*BF, j*_	Façade area of the internal buildings of UrbanTile_ j	*A*_*BF*_	Sum of *A*_*BF, j*_ at the patch scale
*A*_*V, j*_	Footprint area of the internal vegetation objects of UrbanTile_ j	*A*_*V*_	Sum of *A*_*V, j*_ at the patch scale
*AH*_*B, j*_	Product of the internal building height and footprint area of UrbanTile_ j	*G*_*C, i*_	Footprint geometry of the circular area of D-radius of corresponding UrbanTile_i
*AH*_*B*_	Sum of *AH*_*B, j*_ at the patch scale	*G*_*T, j*_	Footprint geometry of UrbanTile_ j
*G*_*S, z*_	Footprint geometry of Street_z	*H*_*B*_	Average building height
*D*_*B*_	Building density	*D*_*V*_	Vegetation density
*VH*_*B*_	Vertical-to-horizontal ratio		

Note: *i* is the index of the target UrbanTile instance; its constructed UrbanPatch area based on D-radius is *G*_*C, i*_; *j* is the index of an arbitrary UrbanTile instance; UrbanPatch_j represents any UrbanTile instances that intersect with *G*_*C, i*_; *z* is the index of arbitrary Street instances.

With UPTO, this study constructed a tiled dataset that composes of thirty major cities in the United States. In this dataset, the UrbanPatch instances are generated based on UrbanTile, and the default D-radius is 500 m. The raw geometry information of the objects (buildings, vegetation, or streets) was collected from OpenStreetMap(https://www.openstreetmap.org/), which follows the Open Data Commons Open Database License (ODbL). All raw information is freely available for reproduction, distribution, transmission, and adaptation. Also, the target of generation can be “Building Objects”, and the D-radius can be changed based on the users’ needs. For generating a new dataset, “Code Availability” section provides more detailed instructions.

### Data serialization

Instances of all UPTO classes are expressed with RDF statements in the triple form of (subject, predicate, object), for example, (“upto:UrbanPatch_0”, “upto:containsTile”, “upto:UrbanTile_0”). RDF represents information as graphs and is understandable to human users, however, such data format is difficult for machines to process. Then the triple-form data need to transform as a structuralized dataset, and this process is called serialization. This study adopted the Python package RDFLib (https://rdflib.readthedocs.io/) to serialize RDF data models into .TTL (Turtle Syntax) files. Turtle is a textual syntax for RDF, which allows RDF graphs to be completely written in a compact and natural text form.

## Data Records

The final tiled multi-city urban objects dataset can be accessed with Figshare^20^. Each city in the dataset is saved as a separate TTL file. Table 2. lists the statistics of the dataset.

**Table 2:** Statistics of tiled multi-city urban objects dataset based on UPTO.

Index	City	Number of Objects			Number of UrbanTiles	Number of UrbanPatches	File Size (MB)
		Building	Vegetation	Street			
1	Albuquerque	198,583	2,634	49,775	10,262	10,262	241
2	Atlanta	59,176	967	17,171	2,475	2,475	72
3	Austin	317,359	4,163	55,832	9,754	9,754	312
4	Charlotte	162,267	3,523	38,318	5,495	5,495	162
5	Chicago	825,739	12,345	139,141	25,823	25,823	1034
6	Columbus	142,007	11,027	79,667	11,812	11,812	300
7	Dallas	345,730	4,957	108,742	20,915	20,915	492
8	Denver	175,456	53,878	55,599	9,314	9,314	291
9	Detroit	599,923	19,611	265,668	43,052	43,052	1075
10	Fort Worth	251,540	2,100	53,290	12,210	12,210	285
11	Houston	170,861	5,313	108,886	23,067	23,067	397
12	Indianapolis	125,167	4,041	63,878	9,300	9,300	222
13	Jacksonville	83,250	3,644	57,916	9,113	9,113	195
14	Las Vegas	70,612	6,186	109,159	18,866	18,866	310
15	Los Angeles	1,198,643	6,824	136,134	27,996	27,996	1095
16	Nashville	12,125	454	9,871	1,179	1,179	28
17	New York	1,266,307	26,896	220,590	49,201	49,201	1863
18	Oklahoma City	18,510	4,252	40,726	7,953	7,953	114
19	Philadelphia	84,144	5,019	64,986	15,714	15,714	369
20	Phoenix	417,434	4,355	102,555	18,532	18,532	458
21	Portland	135,881	1,504	27,225	5,963	5,963	176
22	Saint Louis	48,960	11,929	97,740	17,149	17,149	286
23	Salt Lake City	209,389	7,977	73,886	10,183	10,183	272
24	San Antonio	34,406	3,553	69,350	14,001	14,001	196
25	San Diego	66,373	4,835	70,228	11,096	11,096	207
26	San Francisco	162,381	4,413	25,531	5,889	5,889	210
27	San Jose	379,203	6,806	77,813	9,220	9,220	412
28	Seattle	224,709	4,576	58,308	9,590	9,590	344
29	Tucson	210,075	4,695	62,132	7,925	7,925	278
30	Washington DC	199,793	6,259	41,552	7,415	7,415	264

The .TTL file contains the predefined classes, properties, and instance data of UPTO. The data can be reorganized as the triple form of (subject, predicate, and object). Each triple presents two resources that are related. The subject and the object are the two resources related to each other, and the predicate represents the content of their relationships. Each .TTL file uses compact URIs (Uniform Resource Identifier) and shortcuts to prevent repeats in triples. For example, if several triples share the same subject, the predicates and objects are listed and separated by semicolons. Tables 3, 4 show the classes, properties, and instances of RDF statement samples stored in a .TTL file.

**Table 3:** Classes and Properties of RDF statement in a .TTL file.

Subject	Predicate	Object (Sample instance)
upto:Building	rdf:type	owl:Class
upto:Vegetation	rdf:type	owl:Class
upto:Street	rdf:type	owl:Class
upto:UrbanTile	rdf:type	owl:Class
upto:UrbanPatch	rdf:type	owl:Class
upto:containsBuilding	rdf:type	owl:ObjectProperty
upto:containsStreet	rdf:type	owl:ObjectProperty
upto:containsTile	rdf:type	owl:ObjectProperty
upto:containsVegetation	rdf:type	owl:ObjectProperty
upto:withinTile	rdf:type	owl:ObjectProperty
upto:withinPatch	rdf:type	owl:ObjectProperty
upto:hasTileNeighbor	rdf:type	owl:ObjectProperty
upto:hasStreetNeighbor	rdf:type	owl:ObjectProperty
upto:hasArea	rdf:type	owl:DatatypeProperty
upto:hasPerimeter	rdf:type	owl:DatatypeProperty
upto:hasHeight	rdf:type	owl:DatatypeProperty
upto:hasBuildingType	rdf:type	owl:DatatypeProperty
upto:hasGeometry	rdf:type	owl:DatatypeProperty
upto:hasAvgBuildingHeight	rdf:type	owl:DatatypeProperty
upto:hasBuildingDensity	rdf:type	owl:DatatypeProperty
upto:hasVegetationDensity	rdf:type	owl:DatatypeProperty
upto:hasVerticalToHorizontalRatio	rdf:type	owl:DatatypeProperty

**Table 4:** Instances of RDF statement in a .TTL file.

Subject	Predicate	Object (Sample instance)
upto:Building_0	rdf:type	upto:Building
	rdf:type	owl:NamedIndividual
	upto: hasArea	35.73
	upto:hasPerimeter	23.95
	upto:hasHeight	3.50
	upto:hasBuildingType	“Office”
	upto:withinTile	upto:UrbanTile_0
	upto:hasGeometry	“POLYGON ((…))”
upto: Vegetation_0	rdf:type	upto:Vegetation
	rdf:type	owl:NamedIndividual
	upto:hasArea	101.91
	upto:hasPerimeter	41.09
	upto:withinTile	upto:UrbanTile_0
	upto:hasGeometry	“POLYGON ((…))”
upto:Street_0	rdf:type	upto:Street
	rdf:type	owl:NamedIndividual
	upto:hasArea	638.42
	upto:hasPerimeter	224.81
	upto:withinPatch	upto:UrbanPatch_0
	upto:hasTileNeighbor	upto:UrbanTile_0
	upto:hasGeometry	“POLYGON ((…))”
upto:UrbanTile_0	rdf:type	upto:UrbanTile
	rdf:type	owl:NamedIndividual
	upto:hasArea	16198.17
	upto:hasPerimeter	533.61
	upto:containsBuilding	upto:Building_0
	upto:containsVegetation	upto: Vegetation_0
	upto:withinPatch	upto:UrbanPatch_0
	upto:hasTileNeighbor	upto:UrbanTile_1
	upto:hasStreetNeighbor	upto:Street_0
	upto:hasGeometry	“POLYGON ((…))”
upto:UrbanPatch_0	rdf:type	upto:UrbanPatch
	rdf:type	owl:NamedIndividual
	upto:containsTile	upto:UrbanTile_0
	upto:containsStreet	upto:Street_0
	upto:hasAvgBuildingHeight	2.99
	upto:hasBuildingDensity	0.12
	upto:hasVegetationDensity	0.06
	upto:hasVerticalToHorizontalRatio	0.10

**Fig. 5:**
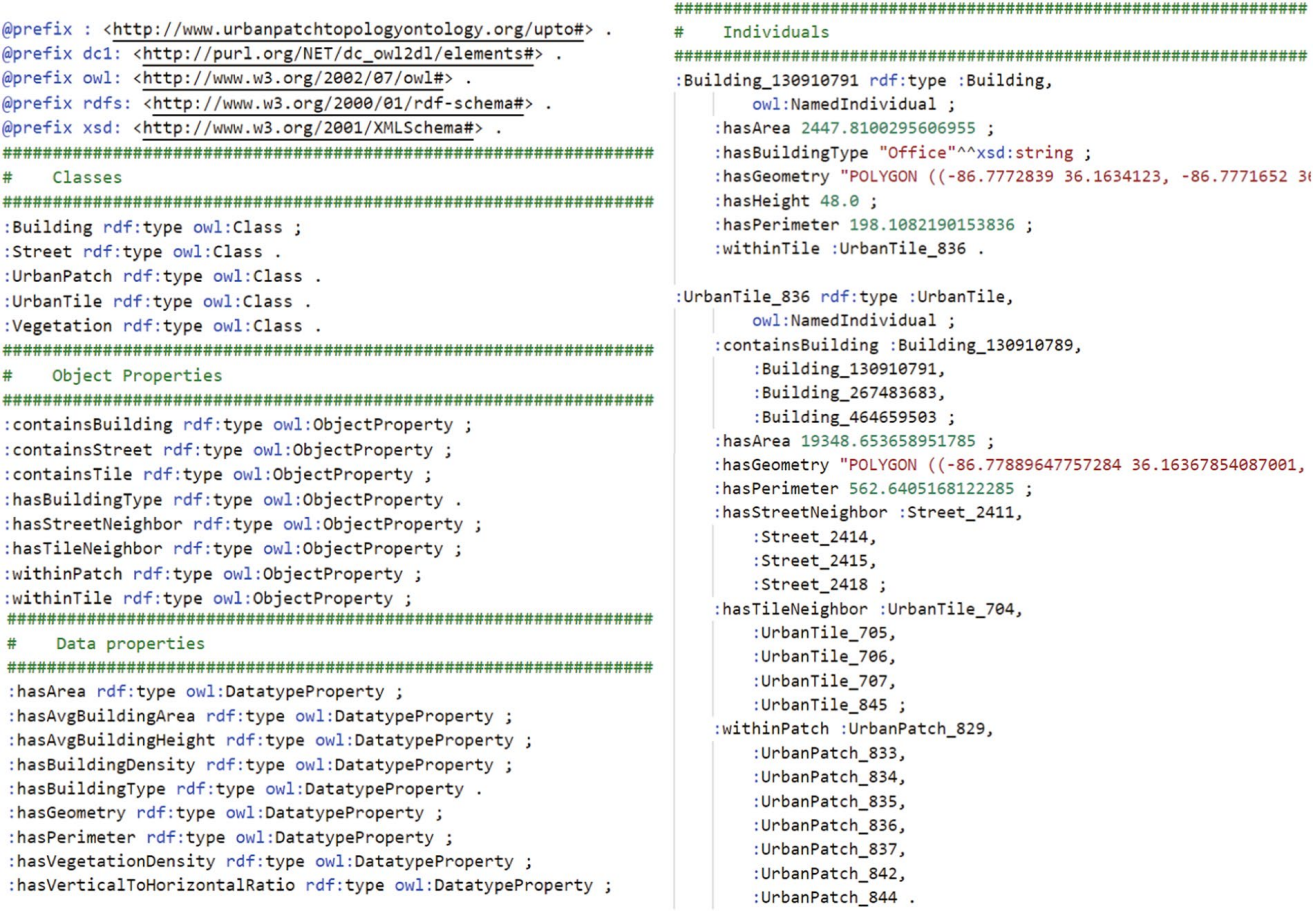
Screenshots of a sample .TTL file.

Figure 5 shows the screenshots of a sample .TTL file, further showing the text data structure of classes, properties, and instances.

Table 5 further lists the data type and unit of objects under each owl:DatatypeProperty. This dataset adopted the geographic coordinate system of EPSG:4326 (WGS84), which is a latitude/longitude coordinate system based on the Earth’s center of mass.

**Table 5:** Data Type and Unit of Objects under Each “owl:DatatypeProperty”.

owl:DatatypeProperty	Data type	Unit
upto:hasArea	float	square meter
upto:hasPerimeter	float	meter
upto:hasHeight	float	meter
upto:hasBuildingType	string	—
upto:hasGeometry	string	—
upto:hasAvgBuildingHeight	float	meter
upto:hasBuildingDensity	float	—
upto:hasVegetationDensity	float	—
upto:hasVerticalToHorizontalRatio	float	—

## Technical Validation

**Fig. 6:**
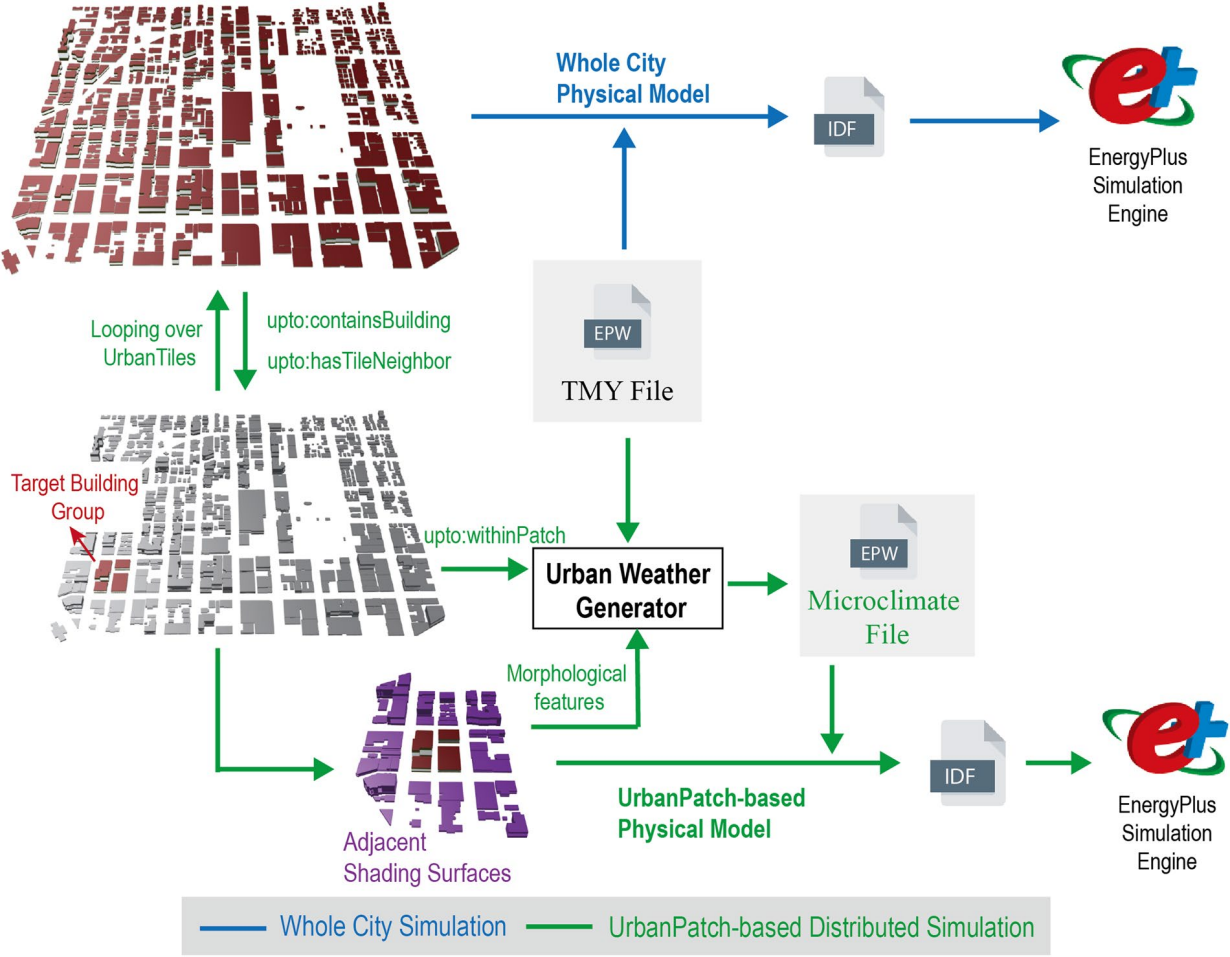
Workflow of the whole city simulation model and UPDS model.

The proposed tiled dataset is designed for large-scale urban building simulation for its scalability in converting the whole city model into UrbanPatches. The conventional simulation model is the whole city simulation model and with the help of UPTO, the simulation can be decomposed into multiple simulation iterations, which is called the UrbanPatch-based Distributed Simulation model (UPDS). Figure 6 further illustrates the workflow of both simulation models. There are two major differences between these two approaches. First, the scale of the simulation. The whole city simulation model simulates the entire city and requires large memory to store the information of building geometric models. It computes all possible interactive thermal exchanges among urban objects. The UPDS model is designed to simulate urban environments at a granular level. It operates by simulating one tile or building at a time and iterating through all tiles or buildings in a given city. In order to capture the thermal interactions between adjacent UrbanTiles, UPDS considers only those with at least one street neighbor. By focusing on these adjacent spatial relations, the model is able to provide accurate thermal simulations. Each iteration of the simulation can handle a small-scale subset of the entire city model, allowing for a comprehensive analysis of the urban environment. Second, the use of micro-climate weather information. The whole city simulation model uses a universal TMY weather file to assess the impact of the external environment. The UPDS model will first calculate a microclimate weather file based on surrounding tile morphological features. Each building/UrbanTile will use a different microclimate weather file during the simulation. A target building and UrbanTile will identify effective adjacent shading surfaces by querying the “hasTileNeighbor” and “containsBuilding” properties of the dataset.

The validation simulation was conducted for the city of Portland. Portland is the largest city in the state of Oregon in the United States. It has a Mediterranean climate with cool, rainy winters and warm, dry summers. All validation simulations were run on a laptop computer with an Intel i5 dual-core central processing unit (Intel Core i5-3427U @ 1.80 GHz), 8 GB of RAM, and a 256 G solid-state hard drive. In order to streamline the simulation model, each building is treated as a single thermal zone, with a fixed window-to-wall ratio (WWR) of 0.5. The physical models are built using the GeomEppy Python package (https://github.com/jamiebull1/geomeppy), while the thermal zone settings are based on the large-office prototype model provided by the U.S. Department of Energy (DOE). Due to limitations in the original data source (OSM) of the dataset, the study only selected one default prototype model to showcase the potential of the dataset for microclimate calculations and energy consumption simulations. Table 6 presents the Construction and HVAC details of the DOE large-office prototype model, while further information about the prototype models can be found in the referenced source (https://www.energycodes.gov/prototype-building-models). The D-radius for UrbanPatch construction of the subsets is 500 meters. It is worth mentioning that the microclimate weather files in this study were prepared by tunning the TMY weather file with the local morphological features extracted from the corresponding UrbanPatch^21^. The computational method implemented the UWG^19^ model, which has been adopted by many existing studies and validated in the cities, including Abu Dhabi^22^, Singapore^23^, Vienna^24^, etc.

**Table 6:** Construction and HVAC information of the DOE large-office prototype model.

	Object	Details
Construction	Exterior walls	8 in. heavy-weight concrete, wall insulation, 0.5 in. gypsum board
	Roof	Roof membrane, roof insulation, metal decking
	Foundation	8” concrete wall; 6” concrete slab, 140 lbs heavy-weight aggregate
	Interior Partitions	2 × 4 uninsulated stud wall
	Internal Mass	6 inches standard wood (16.6 lb/ft²)
HVAC	Heating type	One gas-fired boiler
	Cooling type	Water-source DX cooling coil with fluid cooler
	Pump	Primary chilled water (CHW) pumps
	Cooling Tower Type	Open cooling tower with two-speed fans
	Service Water Heating	One main water heater with storage tank

### Impact of the distributed modeling system

**Fig. 7:**
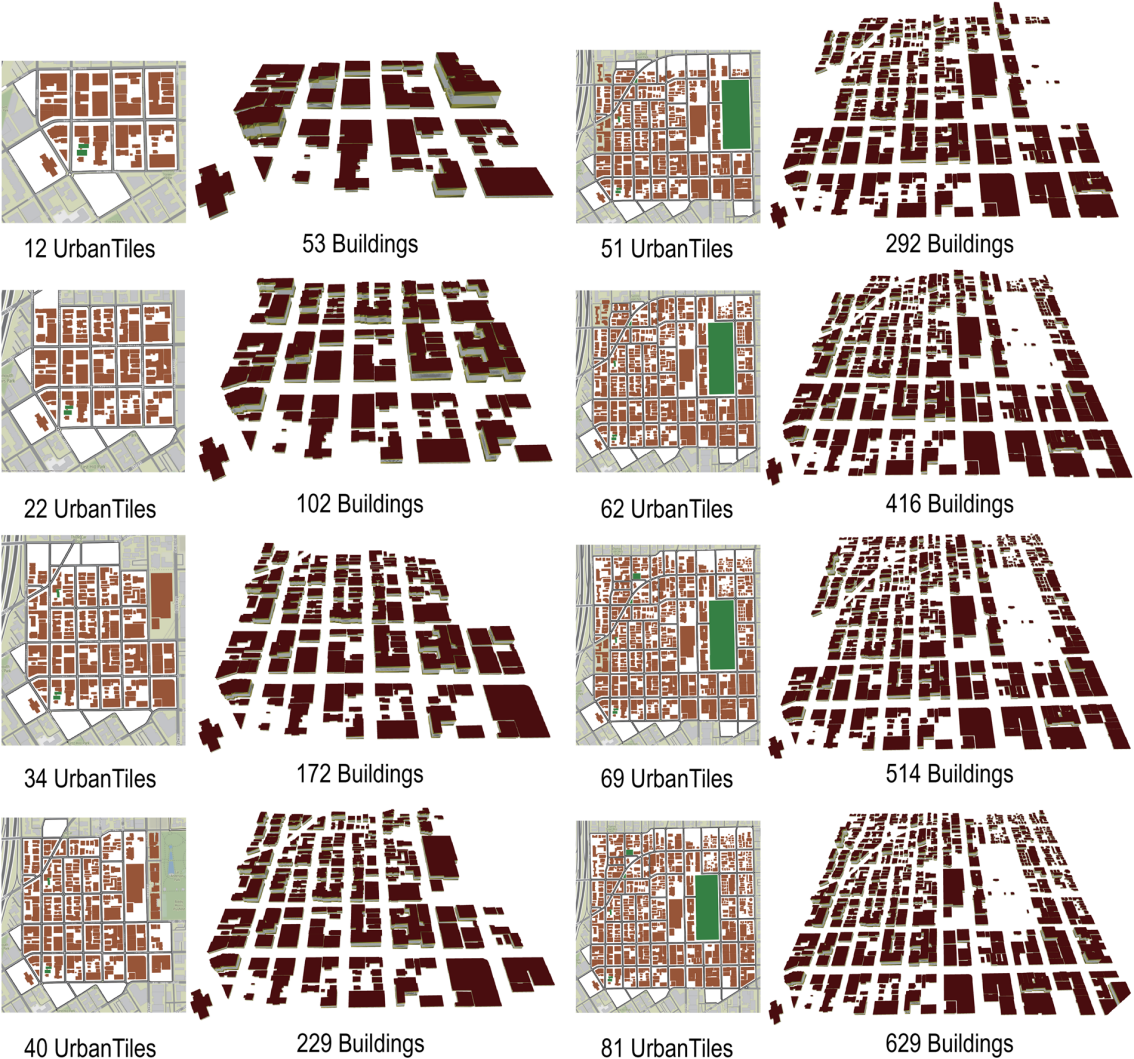
Eight test datasets of different scales in Portland.

To assess the computational efficiency of the developed dataset with the UPDS model, eight subsets with different numbers of buildings and UrbanTiles are selected. Figure 7 shows the 3D building models based on the semantic information of these subsets. The whole city simulation model imported all buildings and objects information as a whole and constructed the geometry and physical model as a single.IDF (Input Data File) file. The UPDS model conducted the simulation iteratively for each tile as a separate.IDF file and aggregated the final results as the final outcomes.

**Fig. 8:**
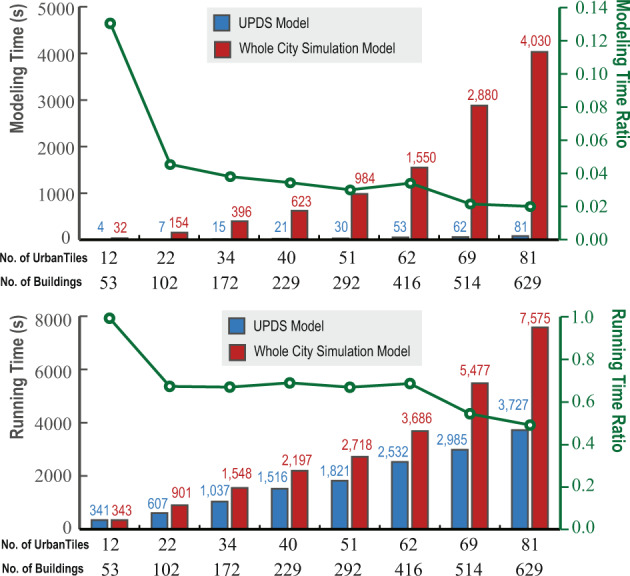
Efficiency comparison between the UPDS model and the whole city model.

The distributed simulation mechanism and abstraction of surrounding physical surfaces are the unique advantages of using this dataset. Figure 8 compares the model construction time and simulation running time for both models. The modeling time of the UPDS model takes much less time compared to the whole city model. With the increase of buildings and UrbanTiles numbers, the model construction time for the whole city simulation model shows a clear exponential trend, whereas the UPDS model has a linear trend. For the running time, with the increase of buildings and UrbanTiles number, both methods show a linear pattern, and the whole city model has a steeper slope. Figure 8 also plots the time ratio of the modeling and running steps. The time ratio is the proportion of the time used by the UPDS model to the time used by the whole city simulation model. It can be seen that with a larger number of objects used from the dataset, the ratio decreases rapidly and tends to converge. Based on the validation set, the converged time ratio for modeling is close to 2% and close to 46% for running. On the one hand, the use of this dataset enables the construction of a distributed physical model at the UrbanTile scale, which is much smaller than the traditional whole city model. This reduces the complexity of the model and makes it possible to simulate more localized and detailed features of the urban environment. On the other hand, the comparison of simulation scenarios was conducted on a single laptop. However, this ontology-based dataset allows for the extraction of relevant information from multiple target UrbanTiles simultaneously, and the calculation tasks can be distributed across multiple computers, significantly reducing the required simulation time.

**Fig. 9:**
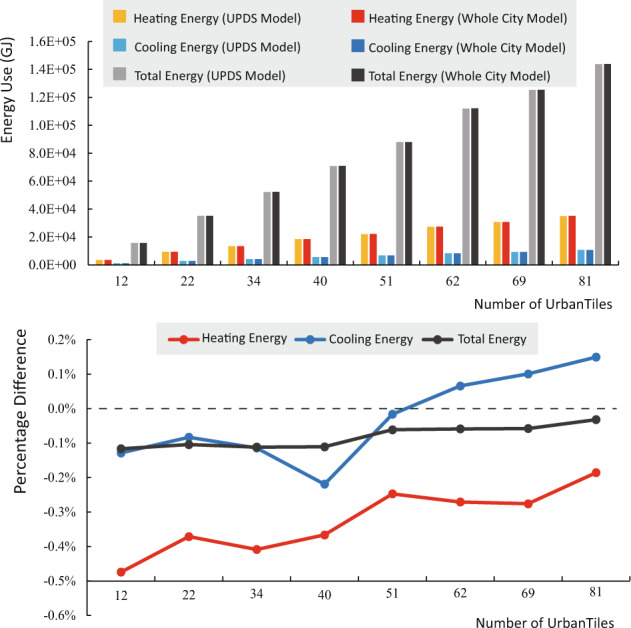
Simulated annual energy use between the UPDS model and the whole city model.

Figure 9 provides a comparison of the annual heating, cooling, and total energy use of the two models. The percentage difference is calculated as the ratio of the change in energy use after using the UPDS model to the result of the traditional whole city model. The results show that the percentage differences in annual heating, cooling, and total energy consumption are negligible. Across the eight testing groups, the maximum absolute value of these three difference indicators does not exceed 0.5%. Interestingly, as the number of UrbanTiles increases, the percentage difference between heating, cooling, and total energy use remains relatively stable. This suggests that the UPDS model designed for this dataset can significantly improve computational efficiency without sacrificing accuracy in the simulation results.

### Impact of microclimates

The UPDS model also has the significant advantage of utilizing high-resolution microclimate weather conditions. To demonstrate how it will differ from conventional single TMY weather, this section conducted a large-scale simulation for 22,448 buildings in 1,392 UrbanTiles. For each target UrbanTile, morphological features can be extracted from its surrounding UrbanPatch.

**Fig. 10:**
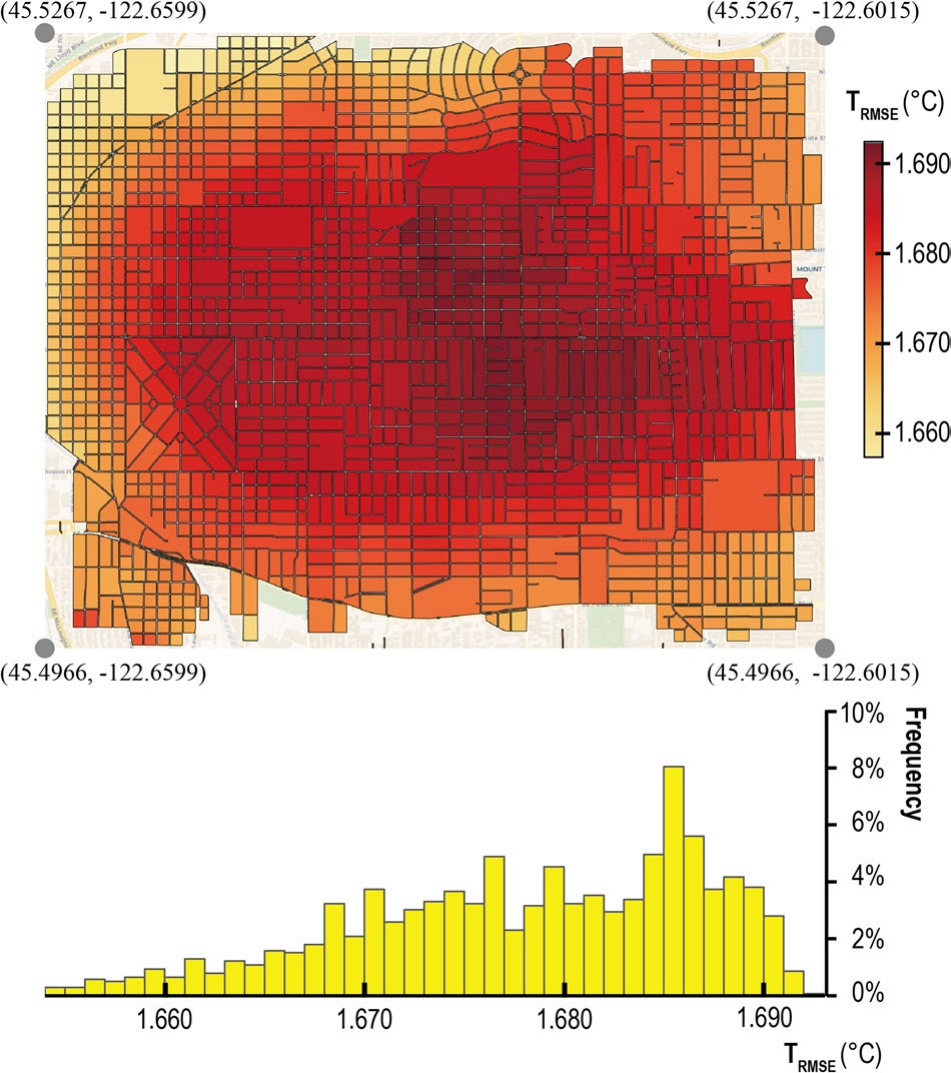
Spatial distribution and histogram of yearly average *T*_*RMSE*_.

Figure 10 presents the annual average root mean square error (RMSE) of temperature *T*_*RMSE*_ when comparing the local microclimate with the TMY weather condition. A *T*_*RMSE*_ value greater than zero for an UrbanPatch indicates that the microclimate within that particular UrbanPatch experiences higher annual hourly average temperatures compared to the suburban weather station. This temperature disparity has significant implications for the cooling and heating energy demands of buildings located in the UrbanPatch. The accompanying histogram highlights that such discrepancies between microclimate and whole city climate conditions are prevalent. Notably, UrbanPatches characterized by denser and taller buildings exhibit larger *T*_*RMSE*_ values, whereas UrbanPatches with more abundant vegetation demonstrate smaller *T*_*RMSE*_ values. In the context of simulating urban building energy consumption, utilizing microclimate data at the UrbanPatch scale offers a more precise depiction of local thermal conditions compared to relying solely on a conventional single TMY weather dataset.”

**Fig. 11:**
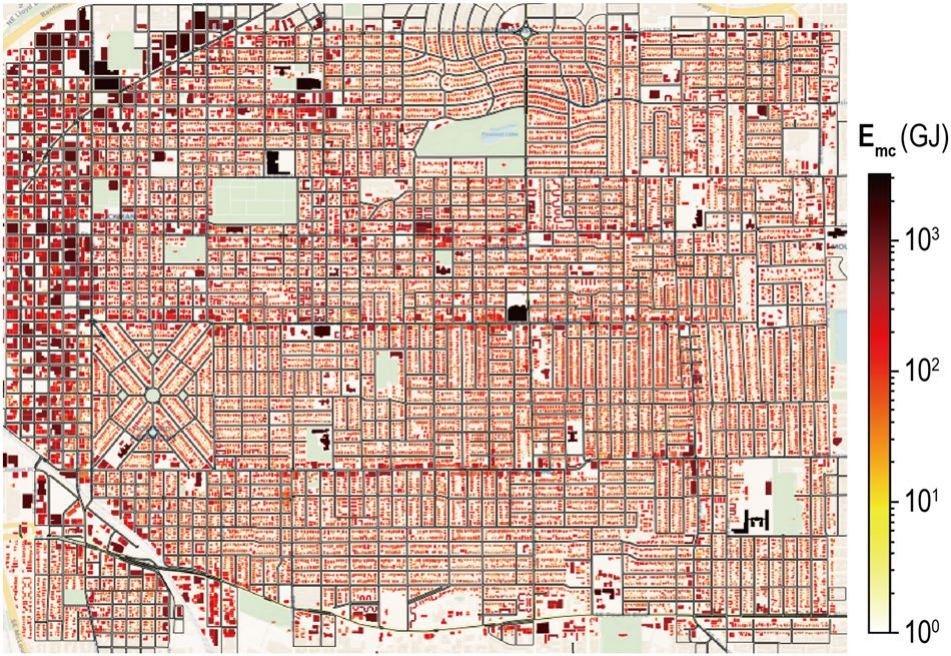
Simulated annual building energy consumptions based on the UPDS model.

The UPDS model conducts building energy simulations for each UrbanTile using distinct microclimate weather files. This simulation process encompasses a total of 1,392 UrbanTiles, enabling all 22,448 buildings within the urban environment to derive their respective energy analysis results. Figure 11 represents the annual total energy consumption of each building, taking into account the prevailing microclimate conditions. Notably, the compact commercial areas located in the northwest corner exhibit higher energy consumption compared to the residential areas. The variable denoted as *E*_*mc*_ represents the energy consumption calculated based on individual microclimate weather conditions. *E*_*mc*_ demonstrates significant variation across different buildings, ranging from 5.33 GJ to 3224.84 GJ. This divergence is influenced by factors such as the building’s footprint area, height, inter-building effects (such as radiation and shading), and other localized environmental factors.

**Fig. 12:**
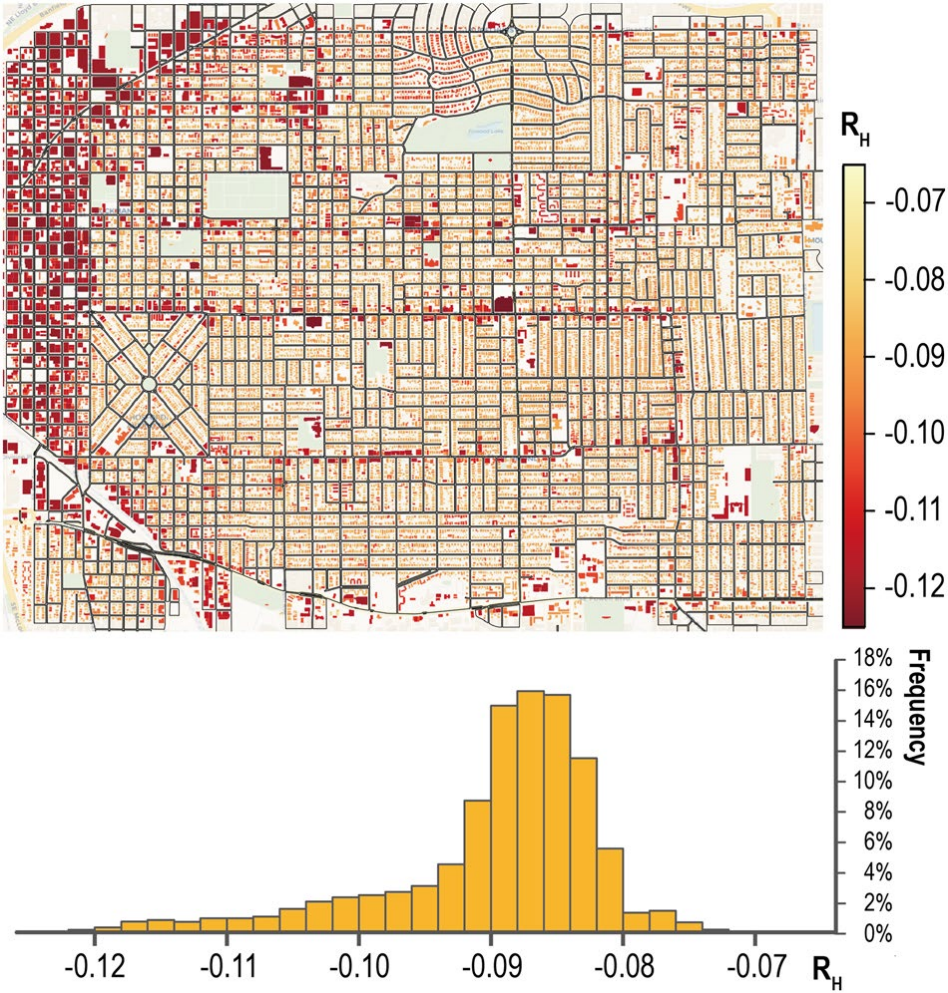
Annual heating load variations between the whole city simulation model and the UPDS model.

**Fig. 13:**
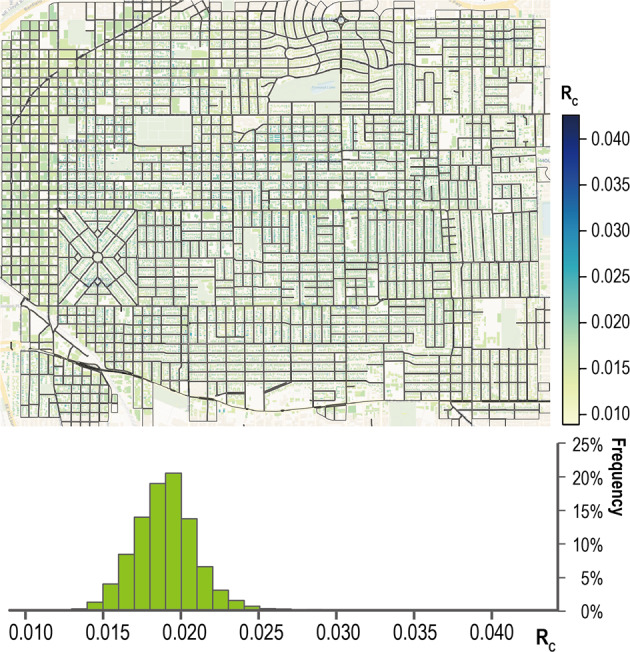
Annual cooling load variations between the whole city simulation model and UPDS model.

Figures 12, 13 further compare the heating energy load variations (*R*_*H*_) and cooling energy load variations (*R*_*C*_) between the whole city simulation model and the UPDS model. A negative value means less energy usage and vice versa. It can be seen that the simulated heating energy load of the UPDS model is lower than that of the whole city simulation model, and that of the cooling load is higher. The absolute value of *R*_*H*_ is larger in locations with high building density. Combining both loads, the highest variation reported in Fig. 11. can reach 66.12GJ. Therefore, it is clear the proposed dataset provides more reliable and simple data sources for large-scale urban building energy simulations. From the perspective of city managers, the simulated results can be used to manage the building stocks and improve retrofit policies and incentives.

The main objective of this dataset is to offer dependable geometry information and microclimate files specifically designed for urban building energy modeling endeavors. The energy and microclimate simulations presented in the study aimed to demonstrate the dataset’s practical application rather than provide an all-encompassing analysis of energy consumption accuracy. It is important to acknowledge that the accuracy of these simulations is contingent upon several factors, including simulation models, underlying assumptions, historical inputs, and other variables.

## Usage Notes

This well-structured semantic dataset allows querying based on description logic and SPARQL (SPARQL Protocol and RDF Query Language). A python package RDFLib is recommended to load .TTL files and provide support for both query methods. For the description logic method, users should filter triple information with the known subjects, predicates, or objects. For example, the internal UrbanTile instance of UrbanPatch_0 can be queried by inputting upto:UrbanPatch_0 (subject) and upto:containsTile (predicate). The SPARQL query method is usually used for complex query conditions, such as querying morphological parameters. Currently, a default UrbanPatch class contains a collection of UrbanTiles and street objects within 500 m. Based on the needs, the UrbanPatch can be constructed for building objects and removing street objects. Also, the D-radius is adjustable to include different sizes of areas. These modifications can be set by defining the object property “containsTile” and “containsStreet”. In each .TTL file, all instance information is stored in a huge directed labeled graph, and RDFLib can be used to add, delete, modify, and query the instance information.

This ontology-based dataset has been specifically designed for the distributed energy simulation of urban buildings. Due to its flexibility, query ability, and machine understandability, it has potential applications in a range of other fields, such as urban-scale or community-scale facility management, IoT information integration, and environmental monitoring. The instances within this dataset can be linked to external ontology-based data through defined relations, expanding its potential applications in other domains. For instance, by linking the building instances in this dataset to Building Product Ontology or Building Automation and Control Systems Ontology instances, one can analyze the spatial distribution of building product information at different scales, from UrbanTile to street scale, or even city scale. This provides new perspectives for facility management. Another example is linking the city objects in this dataset with instances of the Semantic Sensor Network ontology. Depending on the type of sensor, various urban studies can be performed, such as water resource management, air quality or microclimate monitoring, and energy demand assessment. The Semantic Sensor Network ontology is utilized to describe sensors and other properties within the IoT network, while the UrbanTile proposed in this dataset is responsible for describing spatial objects and relationships.

One of the primary advantages of this dataset is its use of semantic web technologies to provide physical entities and microclimate data for distributed energy simulation. This approach enables the dataset to be highly flexible and machine-readable, facilitating easy integration with external ontologies and enabling the data to be easily queried. However, a notable disadvantage of this dataset is that its current data source is OSM, and its data quality largely depends on OSM. While OSM is a valuable resource, it may not always provide the level of accuracy and detail required for certain applications. Therefore, to improve the quality of the dataset over time, it will be necessary to integrate additional data sources and ensure that the data is regularly updated and validated. The OSM data used in this study does not have complete information about building functions. In particular, the upto:hasBuildingType property of building objects is currently set to ‘office’ when performing energy simulations. However, this may not be an accurate representation of the actual building function. We therefore encourage users to update the value of upto:hasBuildingType and choose the corresponding predefined generic models that match the actual building function.

## Data Availability

The shared dataset is prepared based on the default setting of the UrbanPatch container and D-radius. If users want to customizable this dataset with different settings, they can use the shared UrbanPatch generation package (https://github.com/ruirzma/UPTO). There are four files included in the package: • “ConPatchForTile.py”: construct UrbanPatch individuals for UrbanTile objects when changing the receptive radius. • “ConPatchForBuilding.py”: construct UrbanPatch individuals for Building objects for a given receptive radius. • “GenMicroclimate.py”: generate the UrbanTile-scale microclimate. • “GenIDF.py”: generate UrbanTile-scale EnergyPlus IDF file.
